# Common occurrence of divergent *Cryptosporidium* species and *Cryptosporidium parvum* subtypes in farmed bamboo rats (*Rhizomys sinensis*)

**DOI:** 10.1186/s13071-020-04021-5

**Published:** 2020-03-24

**Authors:** Falei Li, Zhenjie Zhang, Suhui Hu, Wentao Zhao, Jianguo Zhao, Martin Kváč, Yaqiong Guo, Na Li, Yaoyu Feng, Lihua Xiao

**Affiliations:** 1 Key Laboratory of Zoonosis of Ministry of Agriculture, College of Veterinary Medicine, South China Agricultural University, Guangzhou, 510642 Guangdong China; 2 Guangdong Laboratory for Lingnan Modern Agriculture, Guangzhou, 510642 Guangdong China; 3 Key Laboratory of Tropical Biological Resources of Ministry of Education, School of Life and Pharmaceutical Sciences, Hainan University, Haikou, 570228 Hainan China; 4 Institute of Parasitology, Biology Centre of the Czech Academy of Sciences, 370 05 Ceske Budejovice, Czech Republic

**Keywords:** *Cryptosporidium*, Zoonotic, Bamboo rat, Subtype, Molecular epidemiology

## Abstract

**Background:**

Bamboo rats are widely farmed in southern China for meat, but their potential in transmitting pathogens to humans and other farm animals remains unclear.

**Methods:**

To understand the transmission of *Cryptosporidium* spp. in these animals, 709 fecal samples were collected in this study from Chinese bamboo rats (*Rhizomys sinensis*) on nine farms in Jiangxi, Guangxi and Hainan provinces, China. They were analyzed for *Cryptosporidium* spp. using PCR and sequence analyses of the small subunit rRNA gene. *Cryptosporidium parvum*, *C. parvum*-like and *C. ubiquitum*-like genotypes identified were subtyped by sequence analysis of the 60 kDa glycoprotein (*gp60*) gene.

**Results:**

Altogether, *Cryptosporidium* spp. were detected in 209 (29.5%) samples. The detection rate in samples from animals under two months of age (70.0%,105/150) was significantly higher than in samples from animals above 2 months (18.6%, 104/559; *χ*^2^ = 150.27, *df* = 1, *P* < 0.0001). Four *Cryptosporidium* species/genotypes were identified: *C. parvum* (*n* = 78); *C. occultus* (*n* = 1); a new genotype that is genetically related to *C. ubiquitum* (*n* = 85); and another new genotype that is genetically related to *C. parvum* (*n* = 44). Among them, *C. parvum* (27,610 ± 71,911 oocysts/gram of feces) and the *C. parvum*-like genotype (38,679 ± 82,811 oocysts/gram of feces) had higher oocyst shedding intensity than the *C. ubiquitum*-like genotype (2470 ± 7017 oocysts/gram of feces) and the *C. occultus* (1012 oocysts/gram of feces). The *C. parvum* identified belonged to three subtypes in two rare subtype families, including IIpA9 (*n* = 43), IIpA6 (*n* = 6) and IIoA15G1 (*n* = 9), while the *C. parvum*-like and *C. ubiquitum*-like genotypes generated very divergent *gp60* sequences.

**Conclusions:**

Results of the present study suggest that bamboo rats on the study farms were infected with diverse *Cryptosporidium* species and divergent *C. parvum* subtypes, which probably had originated from their native habitats. As similar *C. parvum* subtypes have been recently detected in humans and farmed macaques, attentions should be paid to the potential role of these new farm animals in the transmission of zoonotic pathogens.
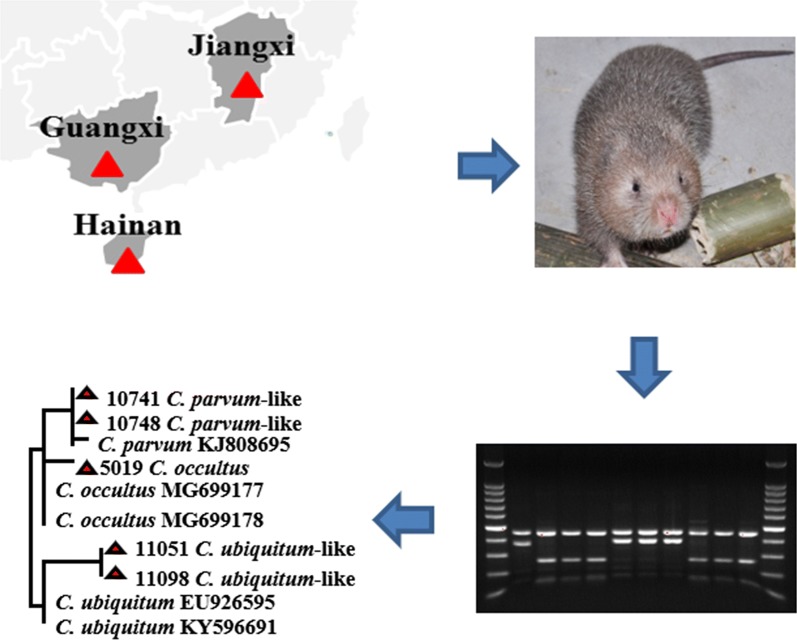

## Background

*Cryptosporidium* spp. are protozoan parasites inhabiting the gastrointestinal epithelium of humans and other vertebrate animals [1]. They are ubiquitous in the environment; humans can be infected with *Cryptosporidium* spp. through contact with infected persons (anthroponotic transmission) or animals (zoonotic transmission) and ingestion of contaminated food (food-borne transmission) or water (water-borne transmission) [2].

To date, over 40 *Cryptosporidium* species have been recognized, together with almost an equal number of genotypes [3]. Among them, *Cryptosporidium parvum* has a broad host range and is the major *Cryptosporidium* species associated with the occurrence of diarrhea in farm animals [4]. As one of the two dominant *Cryptosporidium* species in humans, it is an important zoonotic pathogen [2]. Sequence analysis of the 60 kDa glycoprotein (*gp60*) gene has identified over 20 subtype families of *C. parvum* [5]. Among them, the common ones are host-adapted, such as IIa in dairy cattle, IIc in humans, and IId in small ruminants [3]. Others, such as the newly identified subtype families IIp and IIo, were found mainly in bamboo rats and crab-eating macaques [6, 7]. *Cryptosporidium ubiquitum* is another zoonotic species with a broad host range. Sequence analysis of the *gp60* gene has also identified host-adapted subtype families within the species, some of which have been found in humans and small ruminants in industrialized nations, while others have been found in rodents [8]. Therefore, genetic characterization is important in the assessment of the pathogenicity and public health potential of *Cryptosporidium* spp. in animals.

Bamboo rats are widely farmed in China since 1990 due to the high protein content and perceived medical values of the meat [9]. There were about 10,000 farms (households) of bamboo rats in China in 2011, with an annual production of 30 million animals, of which ~ 500,000 were exported to Southeast Asian countries [10]. However, in China, bamboo rats have long been known as a reservoir of the opportunistic pathogen *Penicillium marneffei* [11, 12]. In recent years, other emerging pathogens such as Akabane virus, beta-lactam resistant *Escherichia coli*, *Enterocytozoon bieneusi* and *Giardia duodenalis* have been detected in farmed bamboo rats [9, 13–15]. In a study of 92 fecal samples collected from a pet market in Sichuan Province, those from one asymptomatic and two diarrheic bamboo rats were positive for *C. parvum* [6]. Therefore, as recently domesticated rodents, bamboo rats have the potential of transmitting zoonotic pathogens to other farm animals and humans.

In this study, we examined the occurrence of *Cryptosporidium* spp. in farmed bamboo rats in southern China and identified the presence of diverse *Cryptosporidium* species and divergent *C. parvum* subtypes in these animals. We postulate that these unusual *Cryptosporidium* spp. probably originated from their native habitats.

## Methods

### Specimens

Between September 2017 and December 2018, 709 fecal samples were collected from Chinese bamboo rats (*Rhizomys sinensis*) on nine farms in Jiangxi, Guangxi, and Hainan provinces, China. Most of the farms sampled were newly established with predominantly adult animals and a small number of young animals. In contrast, Farms 1 and 4 were established facilities, had over 1000 bamboo rats per farm, and provided animals to other farmers because of the availability of large numbers of young animals. On these farms, 5–10 bamboo rats were kept in the same pen, except for breeding pairs, which were kept in individual pens. For young animals under 6 months of age, 2–4 samples of fresh fecal pellets were collected from different locations in the pen to minimize repeated sampling of the same animal, while for older animals, only one sample was collected per pen. The animals in the study were divided into 6 convenient age groups: 1–2 months-old; 3–4 months-old; 5–6 months-old; 7–9 months-old; and 1–3 years-old; with a few of unknown age (Table 1). These fecal samples were stored in 2.5% potassium dichromate before DNA extraction.

**Table 1:** Distribution of *Cryptosporidium* species/genotypes and *Cryptosporidium parvum* subtypes in bamboo rats on farms in Jiangxi, Hainan and Guangxi provinces, China

Location	Farm	Animal age	*n*	No. positive (%)	*Cryptosporidium* spp.				
					*C. ubiquitum*-like	*C. parvum*-like	*C. occultus*	*C. parvum*	*C. parvum* subtype
Guangxi	1	0–2 months	9	7 (77.8)	4	3	–	–	–
		7–9 months	6	2 (33.3)	2	–	–	–	–
		1–3 years	88	16 (18.2)	5	4	–	7	IIoA15G1 (*n* = 6)
		Subtotal	103	25 (24.3)	11	7	–	7	IIoA15G1 (*n* = 6)
	2	0–2 months	11	2 (18.2)	1	–	–	1	IIpA6 (*n* = 1)
		1–3 years	43	1 (2.3)	–	–	–	1	–
		Subtotal	54	3 (5.6)	1	–	–	2	IIpA6 (*n* = 1)
	3	0–2 months	4	1 (25.0)	–	–	–	1	IIpA6 (*n* = 1)
		1–3 years	24	2 (8.3)	2	–	–	–	–
		Subtotal	28	3 (10.7)	2	–	–	1	IIpA6 (*n* = 1)
	4	0–2 months	50	34 (68.0)	2	21	–	11	IIpA9 (*n* = 7)
		3–4 months	25	12 (48.0)	10		–	2	IIpA9 (*n* = 1)
		4–6 months	28	11 (39.3)	8	3	–	–	–
		7–9 months	16	5 (31.3)	2	2	–	1	–
		1–3 years	123	6 (4.9)	4	1	–	1	IIpA9 (*n* = 1)
		Subtotal	244	68 (27.9)	26	28	–	14	IIpA9 (*n* = 9)
	5	1–3 years	30	2 (6.7)	1	–	–	1	–
		Subtotal	30	2 (6.7)	1	–	–	1	–
	6	1–3 years	18	2 (11.1)	–	1	–	1	–
		Subtotal	18	2 (11.1)	–	1	–	1	–
Jiangxi	7	0–2 months	18	15 (83.3)	9	–	–	6	IIpA9 (*n* = 3), IIpA6 (*n* = 3)
		3–4 months	19	10 (52.6)	8	–	1	1	IIpA9 (*n* = 1)
		4–6 months	21	7 (33.3)	6	–	–	1	IIpA9 (*n* = 1)
		7–9 months	13	3 (23.1)	3	–	–	–	–
		1–3 years	82	16 (19.5)	12	–	–	4	IIpA9 (*n* = 4)
		Subtotal	153	51 (33.3)	38	–	1	12	IIpA9 (*n* = 9), IIpA6 (*n* = 3)
Hainan	8	0–2 months	17	13 (76.5)	5	4		4	IIpA6 (*n* = 1), IIoA15G1 (*n* = 2)
		3–4 months	6	5 (83.3)	–	4	–	1	IIoA15G1 (*n* = 1)
		4–6 months	3	2 (66.7)	–	1	–	1	–
		Subtotal	26	20 (76.9)	5	9	–	6	IIpA6 (*n* = 1), IIoA15G1 (*n* = 3)
	9	0–2 months	41	34 (82.9)	–	–	–	34	IIpA9 (*n* = 25)
		Unknown	12	1 (8.3)	1	–	–	–	–
		Subtotal	53	35 (66.0)	1	–	–	34	IIpA9 (*n* = 25)
Total		–	709	209 (29.4)	85	45	1	78	IIoA15G1 (*n* = 9), IIpA9 (*n* = 43), IIpA6 (*n* = 6)

### Detection, genotyping and subtyping of *Cryptosporidium* spp.

Aliquots of 200 mg fecal samples were washed to remove potassium dichromate with distilled water by centrifugation at 2000×*g* for 10 min. DNA was extracted from washed fecal materials using the Fast DNA Spin Kit for Soil (MP Biomedical, Santa Ana, CA, USA) as previously described [16]. The extracted DNA was analyzed for *Cryptosporidium* spp. using a nested PCR targeting a ~ 830-bp fragment of the small subunit rRNA (*SSU* rRNA) gene [17]. Representative *Cryptosporidium* species/genotypes were characterized by restriction fragment length polymorphism (RFLP) analysis of the secondary *SSU* rRNA PCR products using restriction enzymes *Ssp*I (New England BioLabs, Massachusetts, USA) and *Vsp*I (Promega, Madison, WI, USA) [17]. The *C. parvum*, *C. parvum-*like genotype and *C. ubiquitum*-like genotype identified in this study were further subtyped by PCR and sequence analysis of the *gp60* gene [18, 19]. The intensity of oocyst shedding was assessed by using a SYBR Green-based qPCR (18S-LC2) targeting a ~ 278-bp fragment of the *SSU* rRNA gene [20]. The master mix of the qPCR contained 10 μl of 2× SYBR Green real-time PCR master mix (Thermo Fisher Scientific, Waltham, MA, USA) in a 20 μl reaction. The qPCR was performed on a LightCycler 480 II (Roche, Indianapolis, IN, USA) as described previously [7]. All qPCR analyses included one positive control and two negative controls. The number of oocysts per gram of feces (opg) was calculated based on the Cq values of the amplification obtained from the analyzed sample against a standard curve generated from qPCR analysis of fecal samples spiked with known numbers of oocysts of the *C. parvum* IOWA isolate (Waterborne, Inc., New Orleans, USA).

### Sequence analysis

All positive PCR products of the *SSU* rRNA and *gp60* genes were sequenced bi-directionally on an ABI 3730 Autosequencer (Applied Biosystems, Foster City, CA, USA) to identify the *Cryptosporidium* species and *C. parvum* subtypes presented, respectively. The nucleotide sequences generated were assembled using ChromasPro 2.1.5.0 (http://technelysium.com.au/ChromasPro.html), edited using BioEdit 7.1.3.0 (http://www.mbio.ncsu.edu/BioEdit/bioedit.html), and aligned with reference sequences from GenBank using ClustalX 2.0.11 (http://clustal.org). The maximum likelihood analysis implemented in Mega 6.0 (http://www.megasoftware.net) was used to assess the phylogenetic relationship of the novel *Cryptosporidium* genotypes to other *Cryptosporidium* species and genotypes. The general time reversible model was used in the phylogenetic analysis, with the robustness of clade formation being assessed using bootstrap analysis with 1000 replicates. Representative nucleotide sequences generated in this study were submitted to the GenBank database under accession the numbers MK956928–MK956937, MK955996–MK956002, MT019967 and MT019968.

### Statistical analysis

*Cryptosporidium* detection rates in bamboo rats were compared among age and reproduction groups using the Chi-square test implemented in SPSS v.20.0 (IBM Corp., New York, NY, USA). Differences were considered significant at *P* < 0.05.

## Results

### *Cryptosporidium* infection in bamboo rats

Of the 709 samples collected from bamboo rats on 9 farms, 209 (29.5%) were positive for *Cryptosporidium* spp. in PCR analysis of the *SSU* rRNA gene. The detection rates in bamboo rats ranged from 5.6% to 76.9% among the 9 farms (Table 1). Farms 8 and 9 in Hainan had significantly higher detection rates than other farms (*χ*^2^ = 17.6, *df* = 1, *P* < 0.0001; *χ*^2^ = 17.3, *df* = 1, *P* < 0.0001; respectively). Among the 6 farms in Guangxi, Farms 1 and 4 had slightly higher detection rates than other farms (*χ*^2^ = 0.866, *df* = 1, *P* = 0.22; *χ*^2^ = 1.62, *df* = 1, *P* = 0.121; respectively). Regarding rat age, the highest detection rate was 70.0% in the 0–2 month-old group, which was significantly higher than in older animals overall (18.6%; *χ*^2^ = 165.2, *df* = 1, *P* < 0.0001), especially in 1–3 year-old animals (11.0%; *χ*^2^ = 194.1, *P* < 0.0001; Table 2).

**Table 2:** Occurrence of *Cryptosporidium* species/genotypes in farmed bamboo rats in Guangxi, Jiangxi and Hainan provinces, China, broken down by age

Age	*n*	No. positive (%)	*Cryptosporidium* spp.				*C. parvum* subtype
			*C. ubiquitum*-like	*C. parvum*-like	*C. occultus*	*C. parvum*	
0–2 months	150	105 (70.0)	21	28	0	56	IIpA9 (*n* = 36), IIpA6 (*n* = 6)
3–4 months	50	28 (56.0)	18	5	1	4	IIpA9 (*n* = 2), IIoA15G1 (*n* = 2)
4–6 months	53	20 (37.7)	14	4	0	2	IIpA9 (*n* = 1), IIoA15G1 (*n* = 1)
7–9 months	36	10 (27.8)	7	2	0	1	–
1–3 years	408	45 (11.0)	24	6	0	15	IIpA9 (*n* = 4), IIoA15G1 (*n* = 6)
Unknown	12	1 (8.3)	1	0	0	0	–
Total	709	209 (29.5)	85	45	1	78	IIpA9 (*n* = 43), IIpA6 (*n* = 6), IIoA15G1 (*n* = 9)

*Abbreviations*: n, total number of samples; –, *gp60* PCR negative

### *Cryptosporidium* species/genotypes

**Fig. 1:**
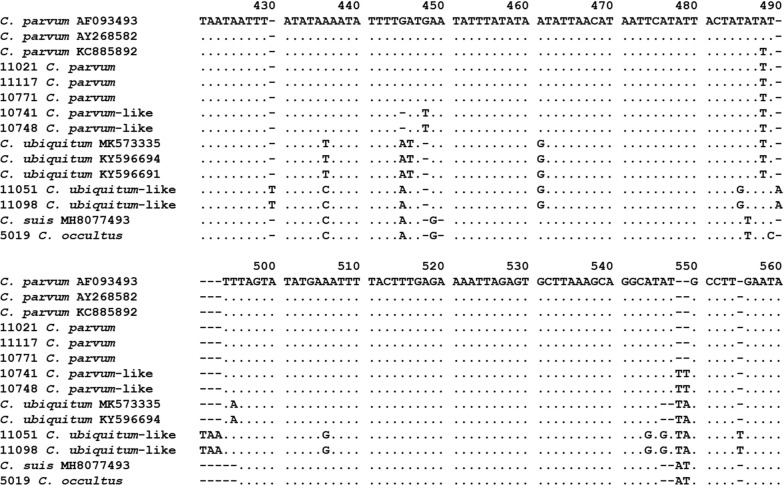
Sequence differences in the hypervariable region of the small subunit rRNA gene among *Cryptosporidium parvum* subtype families (IIa, IIc, IId, IIo and IIp), *C. parvum*-like genotype, *Cryptosporidium ubiquitum*, *C. ubiquitum*-like genotype and *Cryptosporidium occultus*. Dots denote nucleotides identical to those in the GenBank reference sequence AF093493 in the first line, while dashes denote nucleotide deletions

**Fig. 2:**
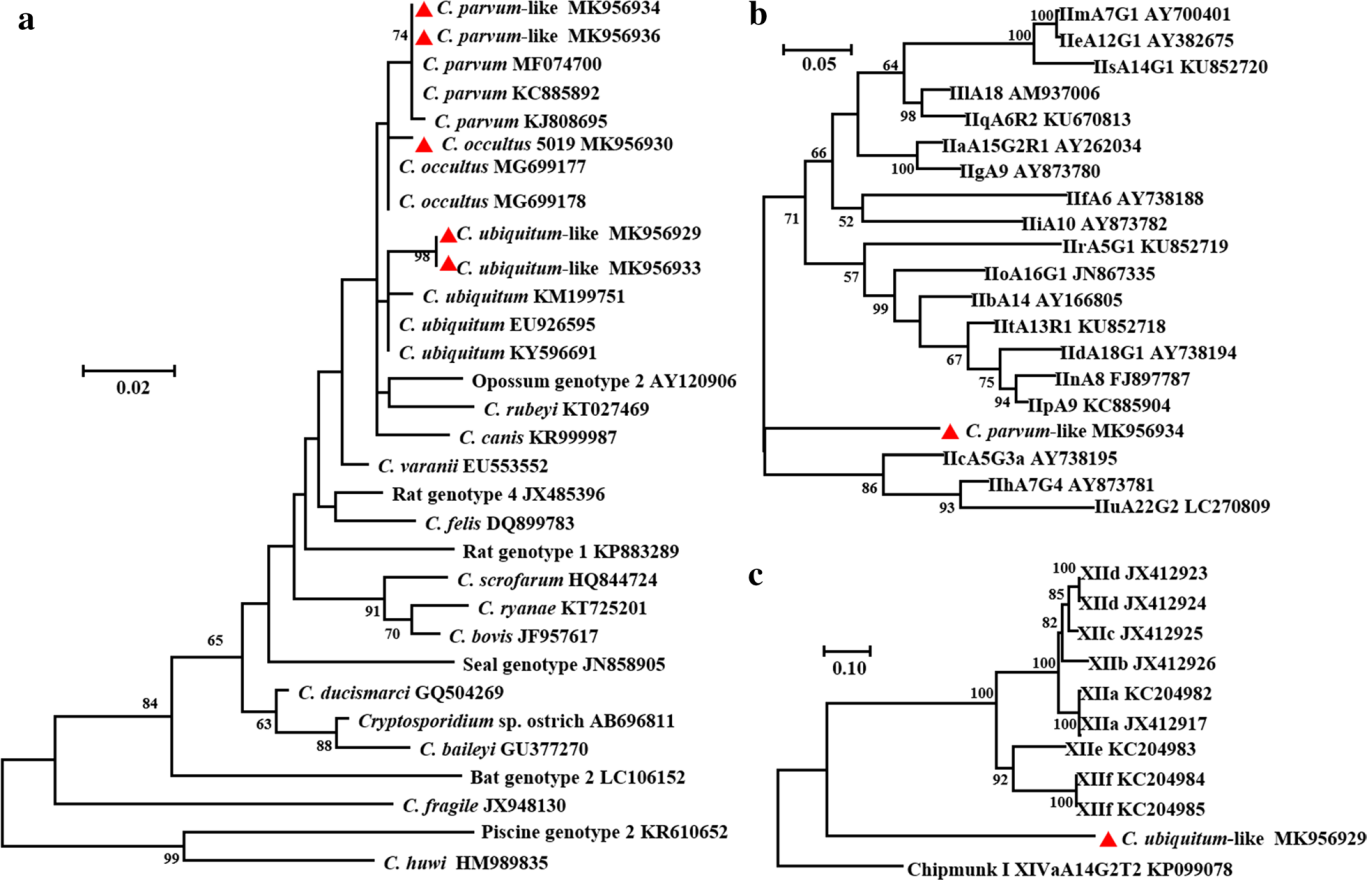
**a** Phylogenetic relationships among *Cryptosporidium* species/genotypes based on the maximum likelihood analysis of the *SSU* rRNA nucleotide sequences. **b** Phylogenetic relationships of *C. parvum*-like genotype and the major *C. parvum* subtype families based on the maximum likelihood analysis of the *gp60* nucleotide sequences. **c** Phylogenetic relationships of *C. ubiquitum*-like genotype and the major *C. ubiquitum* subtype families based on the maximum likelihood analysis of the *gp60* nucleotide sequences. The numbers on the branches are percent bootstrapping values from 1000 replicates, and the sequences generated in the present study are indicated with the red triangles

All 209 *Cryptosporidium*-positive PCR products of the *SSU* rRNA gene were successfully sequenced. The results showed the presence of *C. parvum* (*n* = 78), *Cryptosporidium occultus* (*n* = 1), and two new *Cryptosporidium* genotypes. Of the latter, one was genetically related to *C. ubiquitum* (*n* = 85), while the other was related to *C. parvum* (*n* = 44). The nucleotide sequences generated from *C. parvum* were identical to each other and a nucleotide sequence (GenBank: KC885892) also obtained from bamboo rats [6]. The latter had one A to T substitution from the *SSU* rRNA sequences of *C. parvum* commonly found in humans, cattle and other animals (Fig. 1). Similarly, the nucleotide sequence from *C. occultus* had two nucleotide substitutions compared with the GenBank sequence MH807493 obtained from humans. The *C. ubiquitum*-like genotype had 17 nucleotide substitutions compared with the partial *SSU* rRNA gene sequence obtained previously from *C. ubiquitum* (GenBank: KY596691) in *Chinchilla lanigera* [21], while the *C. parvum*-like genotype had 11 nucleotide differences from a partial *SSU* rRNA gene sequence of *C. parvum* reported from dairy cattle (GenBank: MF074700) [22]. As expected, in the phylogenetic analysis of the *SSU* rRNA nucleotide sequences, the *C. parvum*-like genotype clustered together with *C. parvum*, while the *C. ubiquitum*-like genotype clustered with *C. ubiquitum* (Fig. 2).

**Fig. 3:**
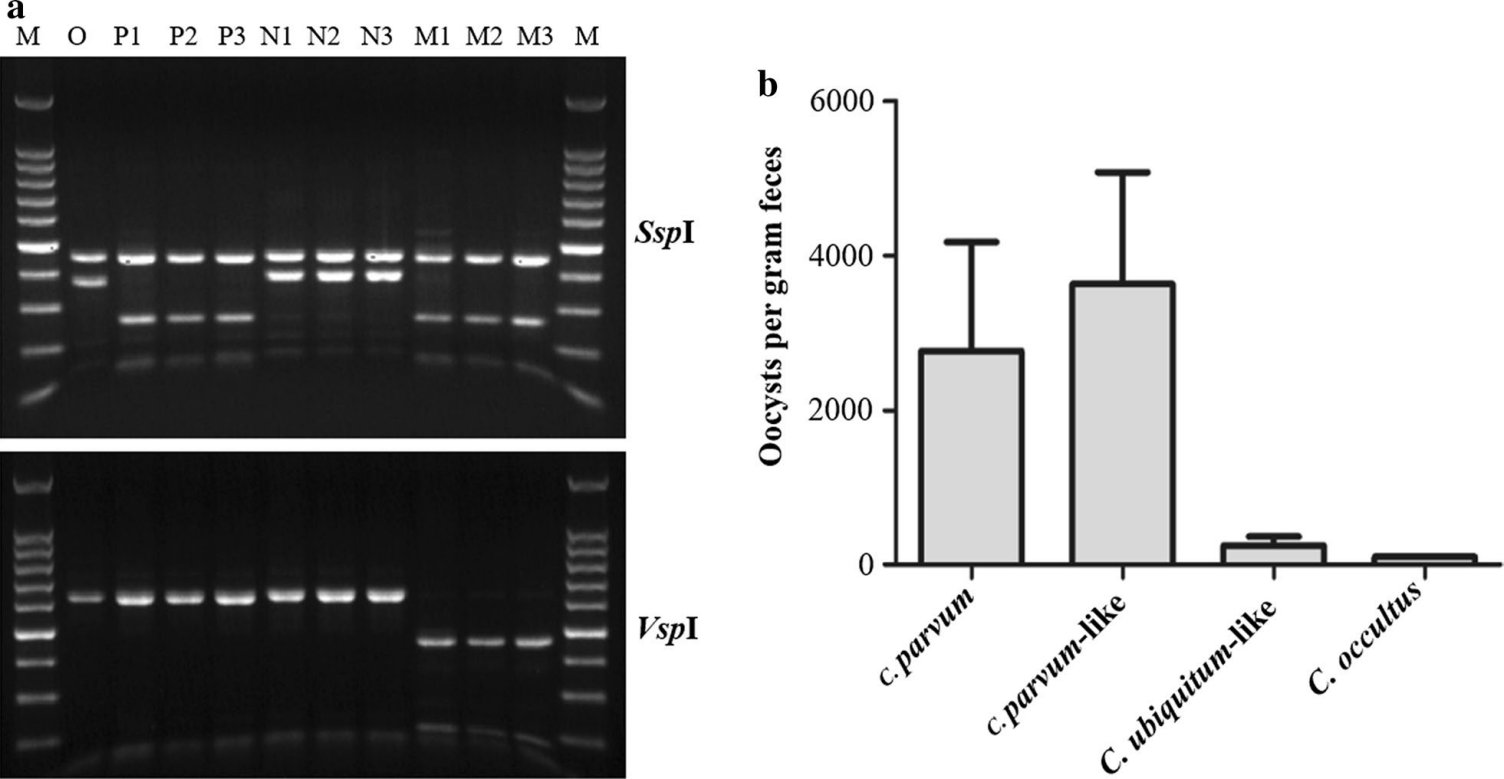
**a** Patterns of restriction fragment length polymorphism analysis of the PCR products of the small subunit rRNA gene in *Cryptosporidium* spp. in bamboo rats. Upper panel: *Ssp*I RFLP patterns; lower panel: *Vsp*I RFLP patterns. Lane M: 100 bp molecular markers; Lane O: *C. occultus*; Lanes P1-P3: *C. parvum*; Lanes N1-N3: *C. ubiquitum-*like genotype; Lanes M1-M3: *C. parvum*-like genotype. **b** Oocyst shedding intensity of *Cryptosporidium* species and genotypes in bamboo rats as indicated by oocysts per gram of feces (mean ± standard deviation)

The *Cryptosporidium* species and genotypes identified in the present study produced different banding patterns in a RFLP analysis of the *SSU* rRNA PCR products using the *Ssp*I and *Vsp*I restriction enzymes. The RFLP profile of *C. occultus* was similar to that of *C. suis*. Similarly, the *C. ubiquitum*-like genotype produced a RFLP profile similar to *C. ubiquitum*. In contrast, the banding pattern for the *C. parvum*-like genotype was different from *C. parvum* due to the presence of a G to A substitution in the hypervariable region of the *SSU* rRNA gene, leading to the creation of an additional *Vsp*I restriction site. This led to the cleavage of the upper *Vsp*I band in *C. parvum* into two smaller fragments (Fig. 3).

### Distribution of *C. parvum*, *C. parvum*-like and *C. ubiquitum*-like subtypes

The 78 *C. parvum*, 45 *C. parvum*-like and 85 *C. ubiquitum*-like isolates were further subtyped by sequence analysis of the *gp60* gene. Among them, 59 of the *C. parvum*, 30 of the *C. parvum-*like and 44 the *C. ubiquitum*-like isolates were successfully subtyped. Three subtypes of two rare subtype families were identified for *C. parvum* samples: IIpA9 (*n* = 43); IIpA6 (*n* = 6); and IIoA15G1 (*n* = 9). One subtype each was identified for *C. parvum*-like and *C. ubiquitum*-like genotypes (Fig. 2). The nucleotide sequences of IIpA9, IIpA6 and IIoA15G1 were identical to the GenBank reference sequence KC885904 obtained from bamboo rats, KC885904 obtained from bamboo rats and JN867335 obtained from humans, respectively [6, 23]. The sequences from the *C. parvum*-like genotype were identical to each other and had a nucleotide identity of 87% to LC270810 obtained from camels [10]. Similarly, the sequences from the *C. ubiquitum*-like genotype had a nucleotide identity of 86% to KX698306 obtained from a water sample [24].

### Oocyst shedding intensity of *Cryptosporidium* spp.

The intensity of oocyst shedding in infected bamboo rats was assessed using 18S-LC2 qPCR. The numbers of oocysts per gram of feces were 27,610 ± 71,911 (*n* = 27), 38,679 ± 82,811 (*n* = 32), 2470 ± 7017 (*n* = 37) and 1012 (*n* = 1) for *C. parvum*, *C. parvum*-like genotype, *C. ubiquitum*-like genotype and *C. occultus*, respectively (Table 1).

## Discussion

Results of this study suggest that *Cryptosporidium* spp. are common in bamboo rats in Jiangxi, Guangxi and Hainan provinces, China. The overall detection rate of 29.5% for *Cryptosporidium* spp. is much higher than the 3.3% (3/92) in the only other study of cryptosporidiosis in bamboo rats conducted from a pet market in Sichuan Province [6]. The intensive nature of animal farming could have contributed to the high prevalence of *Cryptosporidium* spp. in bamboo rats in the present study. As often seen with cryptosporidiosis in other farmed animals, the detection rate was significantly higher in bamboo rats under two months of age (70.0%) than those above 2 months (18.6%). Among the nine farms, *Cryptosporidium* detection rates were higher on the two farms in Hainan, probably because of the sampling of only young animals on these farms. Farms 1 and 4 are leading breeders of bamboo rats in Guangxi. The large size of the farm and frequent animal trade could be responsible for the higher *Cryptosporidium* detection rates (24.3% and 27.9%, respectively) than on the other 4 farms (5.6–11.1%). The higher detection rate of *Cryptosporidium* spp. in breeding animals (13.1%) than in other adults (0–6.7%) could also be attributed to co-housing of animals from different cages.

Altogether, four *Cryptosporidium* species or genotypes were found in bamboo rats in this study. Of these, *C. parvum* has been detected in three bamboo rats previously [6]. The detection of *C. occultus* in one bamboo rat was also expected, as this species is mostly parasitizing rats to which bamboo rats are genetically related [25]. In addition to the two known *Cryptosporidium* species, we showed a common occurrence of two new *Cryptosporidium* genotypes in the studied animals, one genetically related to *C. parvum* and the other related to *C. ubiquitum*. Bamboo rats could be natural hosts of these two new *Cryptosporidium* genotypes, as indicated by their high occurrence in these animals.

The *C. parvum*-like and *C. ubiquitum*-like found in the present study appear to be genetically unique. Although *C. parvum*, *C. ubiquitum*-like and *C. parvum*-like were common in bamboo rats, *C. parvum* and the *C. parvum*-like genotype were mainly detected in animals under two months of age, while the *C. ubiquitum*-like genotype was found in all age groups. In addition, *C. parvum* and the *C. parvum*-like genotype had much greater oocyst shedding intensity than the *C. ubiquitum*-like genotype. This observation is similar to the occurrence of *C. parvum* and *C. ubiquitum* in ruminants [2]. Further studies are needed to understand the host range of the new *C. parvum*-like and *C. ubiquitum*-like genotypes.

*Cryptosporidium parvum* found in bamboo rats in this study belongs to several rare subtypes. This is the most important zoonotic species with a broad host range, including ruminants, equine animals, rodents and primates [26]. However, genetic diversity and host adaptation are known to be present in *C. parvum*, with over 20 subtype families being described by sequence analysis of the *gp60* gene [3]. The IIp subtype family detected in our study was previously reported from only in a few bamboo rats in China [6]. Similarly, the rare *C. parvum* IIo subtype family was first found in diarrheal patients with a history of travel to Thailand [23] and subsequently found in bamboo rats and crab-eating macaques in China [6, 7].

The public health significance of *Cryptosporidium* spp. in bamboo rats is not entirely clear. As mentioned above, the IIo subtype family of *C. parvum* found in the present study appears to be a minor human pathogen that has been found in only a few cryptosporidiosis cases [23]. However, it has recently been reported in 18 farmed crab-eating macaques in China [7]. Therefore, precaution should be taken to prevent the spread of this unique *C. parvum* subtype in farm animals. Similarly, although *C. occultus* has only been found in a few human cases [27], it appears to have a broad host range, including cattle, yak and Tanezumi rats [28–30]. As the new *Cryptosporidium* genotypes identified in this study are genetically related to *C. parvum* and *C. ubiquitum*, two well-known zoonotic *Cryptosporidium* species [3, 8], there is a need to examine their potential as causative agents of human infection.

## Conclusions

Several *Cryptosporidium* species and genotypes, namely *C. parvum*, a *C. parvum*-like genotype, and a *C. ubiquitum*-like genotype, appear to be common in farmed bamboo rats in southern China. The *C. parvum* IIp and IIo subtype families may have initially originated from native rodents, but have recently expanded to humans and non-human primates in China and Southeast Asia. Attention should be paid to monitoring the dispersal of these emerging *C. parvum* subtypes in farm animals.

## Data Availability

Data supporting the conclusions of this article are included within the article. Representative nucleotide sequences generated in the study were submitted to the GenBank database under the accession numbers MK956928-MK956937, MK955996-MK956002, MT019967 and MT019968.

## Abbreviations

PCR: polymerase chain reaction
qPCR: a quantitative PCR
*gp60*: 60 kDa glycoprotein gene
*SSU* rRNA: the small subunit rRNA
RFLP: restriction fragment length polymorphism

## References

[1] Fayer R (2010). Taxonomy and species delimitation in *Cryptosporidium*. Exp Parasitol..

[2] Xiao L (2010). Molecular epidemiology of cryptosporidiosis: an update. Exp Parasitol..

[3] Feng Y, Ryan UM, Xiao L (2018). Genetic diversity and population structure of *Cryptosporidium*. Trends Parasitol..

[4] Santin M (2013). Clinical and subclinical infections with *Cryptosporidium* in animals. N Z Vet J..

[5] Feng Y, Xiao L (2017). Molecular epidemiology of cryptosporidiosis in China. Front Microbiol..

[6] Liu X, Zhou X, Zhong Z, Zuo Z, Shi J, Wang Y (2015). Occurrence of novel and rare subtype families of *Cryptosporidium* in bamboo rats (*Rhizomys sinensis*) in China. Vet Parasitol..

[7] Chen L, Hu S, Jiang W, Zhao J, Li N, Guo Y (2019). *Cryptosporidium parvum* and *Cryptosporidium hominis* subtypes in crab-eating macaques. Parasit Vectors..

[8] Li N, Xiao L, Alderisio K, Elwin K, Cebelinski E, Chalmers R (2014). Subtyping *Cryptosporidium ubiquitum*, a zoonotic pathogen emerging in humans. Emerg Infect Dis..

[9] Tang HB, Chen F, Rao G, Bai A, Jiang J, Du Y (2017). Characterization of Akabane virus from domestic bamboo rat, southern China. Vet Microbiol..

[10] Liu J, Tang CH, Zhou DC, Zeng QB (2011). Current situation and countermeasures of bamboo rat in China. J Hunan Environ-Biolog Polytechnic..

[11] Cao C, Liang L, Wang W, Luo H, Huang S, Liu D (2011). Common reservoirs for *Penicillium marneffei* infection in humans and rodents, China. Emerg Infect Dis..

[12] Huang X, He G, Lu S, Liang Y, Xi L (2015). Role of *Rhizomys pruinosus* as a natural animal host of *Penicillium marneffei* in Guangdong, China. Microb Biotechnol..

[13] Ma X, Wang Y, Zhang HJ, Wu HX, Zhao GH (2018). First report of *Giardia duodenalis* infection in bamboo rats. Parasit Vectors..

[14] Wang H, Liu Q, Jiang X, Zhang Y, Zhao A, Cui Z (2019). Dominance of zoonotic genotype D of *Enterocytozoon bieneusi* in bamboo rats (*Rhizomys sinensis*). Infect Gene Evol..

[15] Zhang H, Li K, Wang Y, Rehman MU, Liu Y, Jin J (2017). Investigation and characterization of beta-lactam resistance in *Escherichia coli* strains isolated from bamboo rats (*Rhizomys sinensis*) in Zhejiang province, China. J Vet Med Sci..

[16] Jiang J, Alderisio KA, Singh A, Xiao L (2005). Development of procedures for direct extraction of *Cryptosporidium* DNA from water concentrates and for relief of PCR inhibitors. Appl Environ Microbiol..

[17] Xiao L, Morgan UM, Limor J, Escalante A, Arrowood M, Shulaw W (1999). Genetic diversity within *Cryptosporidium parvum* and related *Cryptosporidium* species. Appl Environ Microbiol..

[18] Alves M, Xiao L, Sulaiman I, Lal AA, Matos O, Antunes F (2003). Subgenotype analysis of *Cryptosporidium* isolates from humans, cattle, and zoo ruminants in Portugal. J Clin Microbiol..

[19] Guo Y, Cebelinski E, Matusevich C, Alderisio KA, Lebbad M, McEvoy J (2015). Subtyping novel zoonotic pathogen *Cryptosporidium* chipmunk genotype I. J Clin Microbiol..

[20] Li N, Neumann NF, Ruecker N, Alderisio KA, Sturbaum GD, Villegas EN (2015). Development and evaluation of three real-time PCR assays for genotyping and source tracking *Cryptosporidium* spp. in water. Appl Environ Microbiol..

[21] Kellnerova K, Holubova N, Jandova A, Vejcik A, McEvoy J, Sak B (2017). First description of *Cryptosporidium ubiquitum* XIIa subtype family in farmed fur animals. Eur J Protistol..

[22] Cai M, Guo Y, Pan B, Li N, Wang X, Tang C (2017). Longitudinal monitoring of *Cryptosporidium* species in pre-weaned dairy calves on five farms in Shanghai, China. Vet Parasitol..

[23] Insulander M, Silverlas C, Lebbad M, Karlsson L, Mattsson JG, Svenungsson B (2013). Molecular epidemiology and clinical manifestations of human cryptosporidiosis in Sweden. Epidemiol Infect..

[24] Yan W, Alderisio K, Roellig DM, Elwin K, Chalmers RM (2017). Subtype analysis of zoonotic pathogen *Cryptosporidium* skunk genotype. Infect Genet Evol..

[25] Kvac M, Vlnata G, Jezkova J, Horcickova M, Konecny R, Hlaskova L (2018). *Cryptosporidium occultus* sp. n. (Apicomplexa: Cryptosporidiidae) in rats. Eur J Protistol..

[26] Ryan U, Fayer R, Xiao L (2014). *Cryptosporidium* species in humans and animals: current understanding and research needs. Parasitology..

[27] Ong CS, Eisler DL, Alikhani A, Fung VW, Tomblin J, Bowie WR (2002). Novel *Cryptosporidium* genotypes in sporadic cryptosporidiosis cases: first report of human infections with a cervine genotype. Emerg Infect Dis..

[28] Langkjaer RB, Vigre H, Enemark HL, Maddox-Hyttel C (2007). Molecular and phylogenetic characterization of *Cryptosporidium* and *Giardia* from pigs and cattle in Denmark. Parasitology..

[29] Ng-Hublin JS, Singleton GR, Ryan U (2013). Molecular characterization of *Cryptosporidium* spp. from wild rats and mice from rural communities in the Philippines. Infect Genet Evol..

[30] Li P, Cai J, Cai M, Wu W, Li C, Lei M (2016). Distribution of *Cryptosporidium* species in Tibetan sheep and yaks in Qinghai, China. Vet Parasitol..

